# A Deep Learning Model for Identifying the Risk of Mesenteric Malperfusion in Acute Aortic Dissection Using Initial Diagnostic Data: Algorithm Development and Validation

**DOI:** 10.2196/72649

**Published:** 2025-06-10

**Authors:** Zhechuan Jin, Jiale Dong, Chengxiang Li, Yi Jiang, Jian Yang, Lei Xu, Ping Li, Zhun Xie, Yulin Li, Dongjin Wang, Zhili Ji

**Affiliations:** 1 Department of General Surgery Beijing Anzhen Hospital Capital Medical University Beijing China; 2 Beijing Institute of Heart, Lung and Blood Vessel Diseases Beijing Anzhen Hospital Capital Medical University Beijing China; 3 Department of Cardiovascular Surgery Nanjing Drum Tower Hospital Nanjing Medical University Nanjing China; 4 Department of Radiology Beijing Anzhen Hospital Capital Medical University Beijing China; 5 Department of Cardiovascular Surgery Beijing Anzhen Hospital Capital Medical University Beijing China; 6 School of Instrumentation and Optoelectronic Engineering Beihang University Beijing China; 7 Department of Hepatobiliary and Pancreaticosplenic Surgery Beijing Chaoyang Hospital Capital Medical University Beijing China

**Keywords:** acute aortic dissection, mesenteric malperfusion, deep learning, computed tomography angiography, multimodality

## Abstract

**Background:**

Mesenteric malperfusion (MMP) is an uncommon but devastating complication of acute aortic dissection (AAD) that combines 2 life-threatening conditions—aortic dissection and acute mesenteric ischemia. The complex pathophysiology of MMP poses substantial diagnostic and management challenges. Currently, delayed diagnosis remains a critical contributor to poor outcomes because of the absence of reliable individualized risk assessment tools.

**Objective:**

This study aims to develop and validate a deep learning–based model that integrates multimodal data to identify patients with AAD at high risk of MMP.

**Methods:**

This multicenter retrospective study included 525 patients with AAD from 2 hospitals. The training and internal validation cohort consisted of 450 patients from Beijing Anzhen Hospital, whereas the external validation cohort comprised 75 patients from Nanjing Drum Tower Hospital. Three machine learning models were developed: the benchmark model using laboratory parameters, the multiorgan feature–based AAD complicating MMP (MAM) model based on computed tomography angiography images, and the integrated model combining both data modalities. Model performance was assessed using the area under the curve, accuracy, sensitivity, specificity, and Brier score. To improve interpretability, gradient-weighted class activation mapping was used to identify and visualize discriminative imaging features. Univariate and multivariate regression analyses were used to evaluate the prognostic significance of the risk score generated by the optimal model.

**Results:**

In the external validation cohort, the integrated model demonstrated superior performance, with an area under the curve of 0.780 (95% CI 0.777-0.785), which was significantly greater than those of the benchmark model (0.586, 95% CI 0.574-0.586) and the MAM model (0.732, 95% CI 0.724-0.734). This highlights the benefits of multimodal integration over single-modality approaches. Additional classification metrics revealed that the integrated model had an accuracy of 0.760 (95% CI 0.758-0.764), a sensitivity of 0.667 (95% CI 0.659-0.675), a specificity of 0.783 (95% CI 0.781-0.788), and a Brier score of 0.143 (95% CI 0.143-0.145). Moreover, gradient-weighted class activation mapping visualizations of the MAM model revealed that during positive predictions, the model focused more on key anatomical areas, particularly the superior mesenteric artery origin and intestinal regions with characteristic gas or fluid accumulation. Univariate and multivariate analyses also revealed that the risk score derived from the integrated model was independently associated with inhospital mortality risk among patients with AAD undergoing endovascular or surgical treatment (odds ratio 1.030, 95% CI 1.004-1.056; *P*=.02).

**Conclusions:**

Our findings demonstrate that compared with unimodal approaches, an integrated deep learning model incorporating both imaging and clinical data has greater diagnostic accuracy for MMP in patients with AAD. This model may serve as a valuable tool for early risk identification, facilitating timely therapeutic decision-making. Further prospective validation is warranted to confirm its clinical utility.

**Trial Registration:**

Chinese Clinical Registry Center ChiCTR2400086050; http://www.chictr.org.cn/showproj.html?proj=226129

## Introduction

Acute aortic dissection (AAD) is a life-threatening cardiovascular emergency, with an inhospital mortality rate of 10%-20% [1,2]. Approximately 20% of patients with AAD develop end-organ malperfusion [3,4]. Among these, mesenteric malperfusion (MMP) is the most lethal subtypes [5], occurring in 3%-14% of patients with AAD [2,6,7] and exhibiting mortality rates ranging from 13% to 60% [6,8,9]. Despite manifesting through abdominal pain, peritoneal irritation, lactic acidosis, and septic shock, the clinical presentation of MMP often remains obscured by primary aortic pathology [6]. Additionally, the current diagnostic paradigm lacks reliable biomarkers for intestinal ischemia [10]. These challenges delay MMP diagnosis, significantly worsening the prognosis [11,12], as mortality reaches 95.2% in patients who miss surgery and receive only medical therapy [6].

MMP, a subtype of acute mesenteric ischemia (AMI), arises from compromised perfusion in the superior mesenteric artery (SMA) and inferior mesenteric artery (IMA) secondary to AAD. The underlying pathophysiology involves 2 distinct mechanisms [13,14]. Static obstruction develops when the dissection flap extends into mesenteric vessels, causing sustained true lumen collapse, whereas dynamic obstruction results from phasic displacement of the dissecting aortic intimal flap during cardiac cycles, leading to intermittent ostial occlusion of branch vessels. Dynamic obstruction accounts for the majority of clinical cases [15], where imaging findings often exhibit indistinct features due to dynamic hemodynamic fluctuations, thereby posing diagnostic challenges.

Computed tomography angiography (CTA) enables simultaneous evaluation of aortic dissection morphology and intestinal ischemic changes, establishing it as the recommended imaging method for diagnosing MMP [16,17]. Prior studies have identified stenosis of the SMA true lumen and thrombotic occlusion as morphological predictors of MMP [12]. Furthermore, CTA can be used to detect specific signs of intestinal ischemia, including bowel dilatation, bowel wall thinning, abnormal bowel wall enhancement, and pneumatosis intestinalis [18]. However, these studies have mostly been conducted in isolation, with obvious limitations. While vascular morphology is the primary diagnostic basis, approximately 20% of MMP cases lack definitive signs of malperfusion on CTA [11]. Concurrently, CTA demonstrates limited sensitivity for early-stage ischemia before overt intestinal signs manifest [18]. Although integrating imaging data from both the vascular and intestinal systems could enhance risk identification, no existing diagnostic framework incorporates this dual-organ analysis.

Deep learning–based medical image analysis has demonstrated significant potential to address the limitations of conventional analytical methods [19], with recent advancements showing marked efficacy in AAD screening and complication diagnostics [20,21]. However, no prior studies have explored deep learning frameworks for risk stratification of MMP. Given the diagnostic complexity of MMP, which requires comprehensive integration of multimodal clinical data [22], we developed a multimodal neural network–based diagnostic model that integrates abdominal aortic and bowel CTA imaging with key laboratory biomarkers. This model facilitates early detection of individuals at high risk of MMP through initial AAD diagnostic indicators, offering an effective tool for rapid initial screening and preoperative assessment.

## Methods

### Study Design and Datasets

In this retrospective diagnostic study, clinicopathological and CTA imaging data were collected from 525 patients with AAD who were treated across 2 hospitals. Specifically, data from 450 hospitalized patients with AAD at Beijing Anzhen Hospital were collected between January 2015 and June 2022; these patients composed the Anzhen cohort, whose data were used for model training and 5-fold cross-validation. The external validation cohort included 75 patients with AAD (Gulou cohort) who received endovascular or surgical treatment at Nanjing Drum Tower Hospital from January 2019 to December 2022. Both cohorts met the inclusion criteria of CTA-confirmed AAD within 14 days of onset, complete clinicopathological and CTA data, and, for the MMP group, AAD complicated by MMP, whereas patients lacking preoperative CTA, with low-quality images, or with prior gastrointestinal surgery were excluded. In each cohort, non-MMP patients were selected as negative controls via propensity score matching to patients with MMP at a 1:4 ratio on the basis of consultation year and sex, yielding the Anzhen cohort (n=450; 90 MMP, 360 non-MMP) and the Gulou cohort (n=75; 15 MMP, 60 non-MMP). Supplemental Methods and Figure S1 in Multimedia Appendix 1 detail the patient selection process for the training and external validation cohorts.

The diagnosis of MMP was established through dynamic clinical observation and defined by the following criteria [6,12,23]: (1) radiologically confirmed vascular insufficiency: radiological evidence of reduced blood flow in the SMA or IMA must be objectively demonstrated, (2) clinical evidence of impaired bowel viability: symptoms and physical findings indicative of diminished intestinal perfusion, including but not limited to abdominal pain, distension, hematochezia, abdominal tenderness, and rigidity—must manifest during disease progression, and (3) ancillary laboratory markers: elevated serum lactate levels and leukocytosis may support the diagnosis but are not mandatory diagnostic criteria.

Guided by the AAD and AMI guidelines [24,25], we selected laboratory variables for MMP prediction, including inflammation, ischemia, liver function, renal function, coagulation, myocardial injury, electrolytes, and nutritional markers (Table S1 in Multimedia Appendix 1). Variables with >10% missing data were excluded before further analysis. All laboratory tests and CTA images were obtained within 12 hours of the emergency department presentation as the initial preoperative results to ensure data timeliness.

### Ethical Considerations

The Medical Ethics Committee of Beijing Anzhen Hospital Affiliated with Capital Medical University approved the study protocol (KS2023020). The study is registered in the Chinese Clinical Trial Registry (ChiCTR2400086050). Given the retrospective nature of this study, the requirement for obtaining patient informed consent was waived. All patient data have undergone deidentification processing to ensure anonymity. As this study was based on retrospective analysis of existing medical records and did not involve direct participation of human subjects, no specific compensation was provided to participants. This report is written in accordance with the standards for reporting of diagnostic accuracy principles [26].

### Data Preprocessing and Multiorgan Segmentation

After the clinicopathological data were collected, we conducted quality control checks on the missing data of the included patients. We subsequently addressed missing values in clinical variables identified during the model-building process using multiple imputation [27]. To mitigate issues arising from differences in feature scales, we standardized the data prior to model construction.

In addition, to minimize confounding effects from CTA image heterogeneity, we conducted a series of preprocessing steps (Figure S2 in Multimedia Appendix 1). First, all the images were converted from Digital Imaging and Communications in Medicine format to the Neuroimaging Informatics Technology Initiative format to optimize computational efficiency. Uniform resampling was subsequently performed to standardize the spatial resolution to 1×1×5 mm, facilitating manual annotation of the volumes of interest (VOIs).

The VOIs, including the abdominal aorta (including the 3 main branches of the celiac trunk and the primary branches of the SMA and the IMA) and the bowel (including the jejunum, ileum, and colon), were manually labeled by a surgeon (Z Jin, reader 1) with 5 years of diagnostic experience using a 3D Slicer. To validate annotation consistency [28], a second surgeon (YJ, reader 2) independently labeled a randomly selected subset (n=30) of images. Interreader agreement was quantified using the Dice similarity coefficient (DSC) [28], with the entire annotation process adhering to a double-blind principle.

To construct the VOIs segmentation models, the CTA images subsequently underwent initial cropping on the basis of the start and end slices of the abdominal aorta and bowel, while the images were downsampled to 224×224×32 pixels. Using the VOIs segmentation masks as references, additional cropping was applied to focus the deep learning models on anatomically critical regions. On the basis of the VOIs segmentation masks, further cropping was performed to focus the deep learning model on key anatomical regions. The images were further zoomed, padded, and resized to 112×112×32 pixels, ensuring computational efficiency while maintaining the integrity of the key anatomical information. Finally, the CTA images were standardized using specific window width and level settings (aorta: width=600 HU, level=250 HU; bowel: width=400 HU, level=40 HU) to highlight the contours of the specific anatomical structures.

### Development of the Deep Learning Model

**Figure 1:**
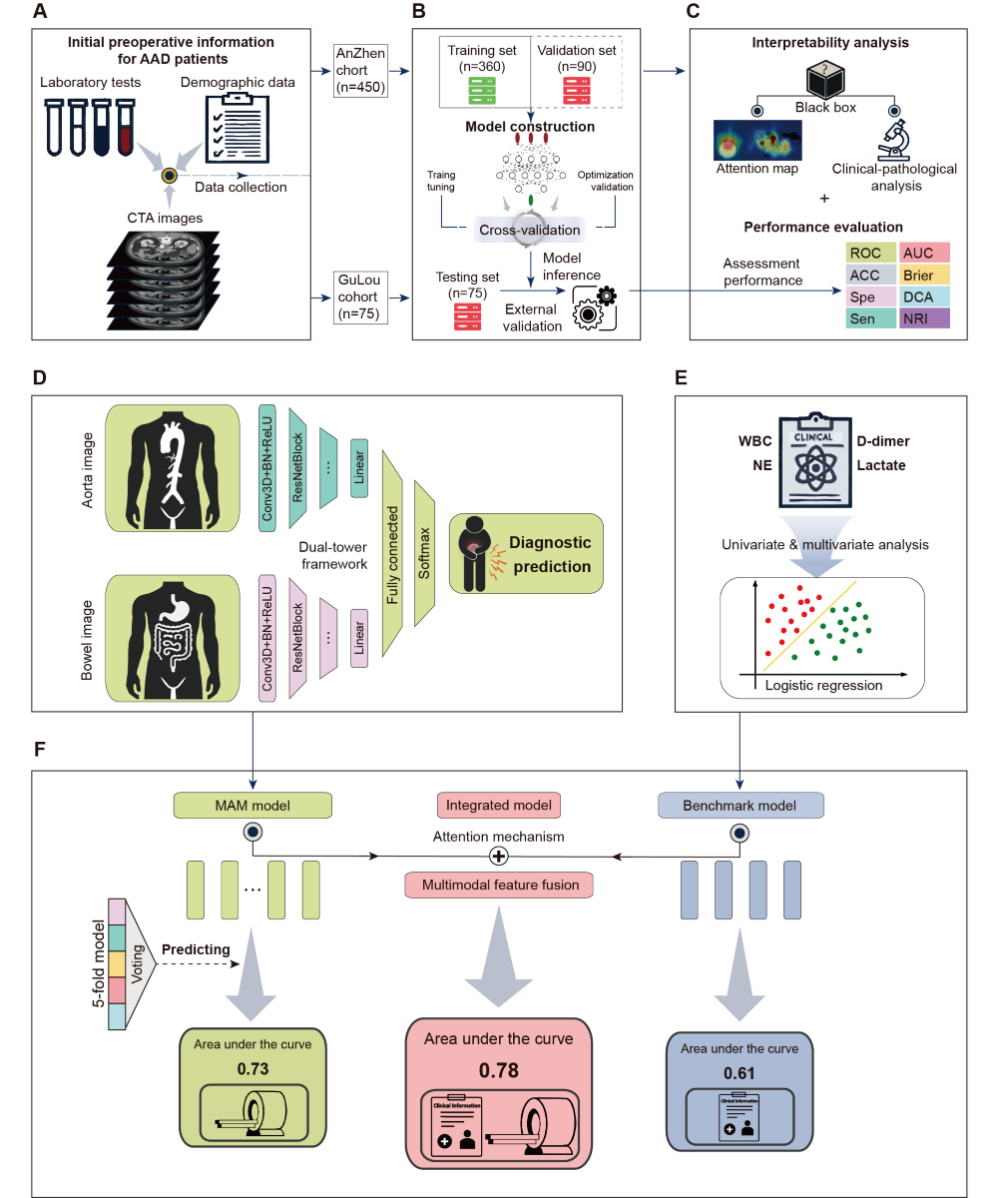
Study process. (A) Demographic data, laboratory tests, and CTA images were collected to construct the Anzhen and Gulou cohorts. (B) The Anzhen cohort served as the training (internal validation) set with 5-fold cross-validation, whereas the Gulou cohort was used as an external validation set to test the overall pipeline performance. (C) Model performance was assessed using ROC curves. The deep learning model was interpreted using gradient-weighted class activation mapping (Grad-CAM) and univariate and multivariate analyses. (D) A dual-tower structured model based on ResNet encoders was trained to predict the diagnosis of AAD complicating mesenteric malperfusion. (E) Independent predictive factors were identified through univariate and multivariate analyses, and a benchmark clinical model was constructed using logistic regression. (F) An integrated model was developed by fusing image features and clinicopathological features using an attention mechanism. AAD: acute aortic dissection; ACC: accuracy; AUC: area under the curve; Brier: Brier score; CTA: computed tomography angiography; DCA: decision curve analysis; NE: neutrophil; NRI: net reclassification index; ROC: receiver operating characteristic; Sen: sensitivity; Spe: specificity; WBC: white blood cell.

The overall research process is depicted in Figure 1. In summary, after collecting the data (Figure 1A), we developed organ segmentation models for the abdominal aorta and bowel using nnU-Net to acquire 3D VOIs of these organs [29]. To accommodate input from multiple-organ VOIs, we developed and tested a multiorgan feature-based AAD complicating MMP (MAM) model (Figure 1D). Concurrently, we constructed a benchmark clinical model on the basis of the fundamental laboratory results at the time of AAD diagnosis (Figure 1E). After establishing the predictive value of both models, we combined the data from these 2 modalities to develop an integrated model (Figure 1F). The specific steps of this study are as follows.

In step 1, to achieve an automated workflow for MMP risk identification, we used the nnU-Net framework to train semantic segmentation models for the abdominal aorta and bowel using CTA images from the Anzhen cohort. Specifically, all the segmentation models were configured with 3D full resolution. Training was conducted with a batch size of 2, an initial learning rate of 0.01 dynamically adjusted with a polynomial learning rate scheduler, stochastic gradient descent optimizer, and combined Dice and cross-entropy loss function, with each model trained for 1000 epochs. To ensure model robustness and reliable evaluation, we adopted a 5-fold cross-validation approach for training and assessment.

For step 2 (Figure 1D), as the diagnosis of AAD with concurrent MMP requires integrating vascular and bowel information, we designed the MAM model with a dual-tower architecture (Figure S3 and Supplemental Methods in Multimedia Appendix 1), featuring 2 independent input pathways to process imaging data from the abdominal aorta and bowel. Specifically, the MAM model uses 2 parallel ResNet10 encoders, each comprising a 3D convolutional layer, a maximum pooling layer, 4 ResNet blocks, and an average pooling layer, to extract features from the respective organs. These features are concatenated in deeper layers and fed into an 8-layer multilayer perceptron for prediction. To address class imbalance and the limited sample size, we combined online data augmentation (including Gaussian noise, contrast adjustment, rotation, and 3D elastic deformation) with weighted random sampling, expanding the training sample size to 500 per epoch. To further reduce overfitting risks, we used Med3D pretrained weights for transfer learning [30] and implemented L2 regularization, batch normalization, dropout, and early stopping. During training, the model incorporated a binary cross-entropy loss function and was optimized using the AdamW algorithm with an initial learning rate of 10^–5^. The learning rate was dynamically adjusted using a learning rate decay strategy based on loss reduction. All the models were trained for 150 epochs with a batch size of 16.

We subsequently performed univariate and multivariate analyses of the associations’ basic laboratory data with the diagnosis and early treatment of AAD. Statistically significant variables were incorporated into a logistic regression model to train a benchmark clinical model (Figure 1E).

Finally, building on the MAM model, we integrated laboratory and imaging data using an attention mechanism to develop an integrated model (Figure 1F) to assess the additional predictive value of these clinicopathological features. All the deep learning models were trained and validated on a hardware setup consisting of 2 NVIDIA RTX 4090 GPUs, an Intel Xeon Gold 6326 CPU at 2.90 GHz, and 256 GB of internal memory. The detailed code and documentation for the deep learning and segmentation models are available on GitHub [31].

### Performance Evaluations

In this study, we used a set of evaluation metrics to assess the performance of the models (Figure 1C). Initially, we calibrated predictive probabilities with 20% of the data from the Anzhen cohort as the calibration set.

Subsequently, the model’s discriminative ability was assessed by calculating the area under the curve (AUC), accuracy, sensitivity, and specificity values, thus providing a comprehensive perspective on the model’s predictive performance. The Brier score was used to represent the consistency between the predicted probabilities and the actual occurrence of MMP [32]. Decision curve analysis was used to evaluate the clinical use of the model, revealing its practical value by calculating the net benefits at various probability thresholds [33]. The net reclassification index was used to assess the improvement in risk classification after the model update through quantification of the enhancement post update [34]. Owing to the limited number of samples and the need to maximize data utilization, we did not establish a separate validation set; instead, we opted for 5-fold cross-validation within the training set (Figure 1B). This strategy allowed us to provide a comprehensive evaluation of model performance through repeated internal training and validation, thus aiding in the selection of hyperparameters.

During the testing and inference phase, we used a voting ensemble of the predictions from the 5 models trained via cross-validation to determine the model’s final output (Figure 1F) [35]. For cutoff setting in model classification, we first determined the optimal thresholds for each model trained during cross-validation using the maximum Youden index [36]; then, the smallest 3 thresholds were averaged for the final classification.

### Clinical Interpretability Analysis

To identify critical areas in computed tomographic images that influence specific predictions, we used gradient-weighted class activation mapping [37], which enhances the understanding of the model’s decision-making process and reveals biological characteristics potentially related to the disease (Figure 1C). This feature also provides clinicians with valuable visual references to aid in diagnostic decisions. Furthermore, to further assess the clinical use of the model, we used the output of the integrated model as a risk score (RS). We subsequently analyzed the impact of this RS, along with other clinicopathological characteristics, on inhospital mortality among patients with AAD undergoing endovascular or surgical treatments.

### Statistical Analysis

Statistical analyses in this study were conducted using SPSS statistical software (version 25; IBM Corp). Continuous variables that are normally distributed are presented as the means with SDs, and those with nonnormal distributions are presented as medians with IQRs. For statistical analysis, the independent samples 2-tailed *t* test was used to compare normally distributed continuous variables, and the Mann-Whitney *U* test was used to compare variables with nonnormal distributions. Categorical variables are presented as numbers with percentages and were compared using the chi-square test or the Fisher exact test. Variables that were significant in univariate logistic regression analyses were subsequently entered into a multivariate logistic regression model to identify independent risk factors for MMP. Multiple imputation methods were used to address missing values and to conduct propensity score matching via R (version 4.1.0; R Core Team). Differences in the AUCs between the models were assessed using the DeLong test for AUC comparisons. Bootstrap resampling with 1000 iterations was performed to estimate 95% CIs for the performance metrics. Differences were considered statistically significant at a *P* value of <.05.

## Results

### Clinical Characteristics

To delineate the clinical characteristics of MMP and assess cohort comparability, we conducted a baseline analysis. A total of 525 patients were included in this study, with the Anzhen cohort (n=450) serving as the training set and the Gulou cohort (n=75) serving as the external validation set. The Anzhen cohort had a median age of 52 (IQR 43-57) years, 91.11% (410/450) of the participants were male, 47.11% (212/450) had type A AAD, and the following chronic comorbidities were detected: hypertension (85.56%), diabetes (4%), and coronary artery disease (11.33%). The Gulou cohort had a median age of 54 (IQR 46-65) years, 86.67% (65/75) of the participants were male, 37.33% (28/75) had type A AAD, and several comorbidities were detected: hypertension (81.33%), diabetes (2.67%), and coronary artery disease (13.33%). Except for an age difference (*P*=.01), no significant differences were observed in the other clinical characteristics between the cohorts (Table 1). Lactate dehydrogenase was excluded because the missing data rate exceeded 10% in the Anzhen cohort (Table S1 in Multimedia Appendix 1). Furthermore, the inhospital mortality rate was 10.44% overall in the Anzhen cohort (Table 1), with the MMP group experiencing approximately twice the mortality rate of the non-MMP group (16.67% vs 8.89%, *P*=.03; Table S1 in Multimedia Appendix 1). Notably, 61 (67.78%) of 90 patients (*P*<.001) presented with abdominal symptoms, and 53 (58.89%) patients (*P*<.001) experienced malperfusion in other organs within the MMP group (Table S1 in Multimedia Appendix 1).

**Table 1:** Characteristics of patients in the Anzhen and Gulou cohorts^a^.

Characteristics			Total (N=525)					*P* value	
			Anzhen (n=450)		Gulou (n=75)				
Age (years), median (IQR)			52 (43-57)		54 (46-65)		.01		
**Sex, n (%)**									.23
	Female	40 (8.89)		10 (13.33)					
	Male	410 (91.11)		65 (86.67)					
**Type of AAD^b^, n (%)**									.11
	Type A	212 (47.11)		28 (37.33)					
	Type B	238 (52.89)		47 (62.67)					
**Inhospital outcome** **, n (%)**									.95
	Survival	403 (89.56)		67 (89.33)					
	Mortality	47 (10.44)		8 (10.67)					
MMP^c^, n (%)			90 (20)		15 (20)		>.99		
**Chronic comorbidities, n (%)**									
	Hypertension	385 (85.56)		61 (81.33)		.34			
	Diabetes	18 (4)		2 (2.67)		.75			
	CAD^d^	51 (11.33)		10 (13.33)		.62			

^a^The values are presented as medians (IQR) for continuous variables and as numbers (%) for categorical variables.

^b^AAD: acute aortic dissection.

^c^MMP: mesenteric malperfusion.

^d^CAD: coronary artery disease.

### Performance of the Segmentation Models

**Figure 2:**
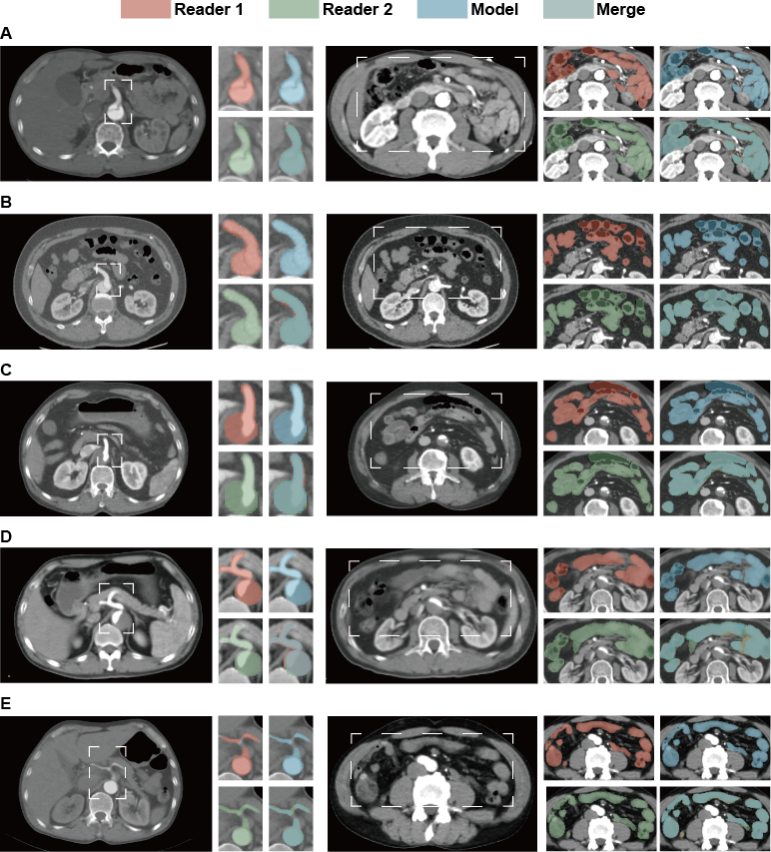
Performance of manual and automatic segmentation. (A-E) Each panel represents a randomly selected sample from the validation sets used during cross-validation, featuring computed tomography angiography images of the abdominal aorta and bowels. Red mask: represents annotations by reader 1. Green mask: represents annotations by reader 2. Blue mask: represents annotations by the deep learning segmentation model. Merge mask: represents the intersection of annotations from all 3 sources (reader 1, reader 2, and the deep learning model).

To automate MMP identification in patients with AAD, we developed CTA-based segmentation models for the abdominal aorta and bowel. The reproducibility analysis of the VOI revealed that the DSCs of the abdominal aorta and bowel were 0.896 (SD 0.026) and 0.907 (SD 0.072), respectively, between reader 1 and reader 2 (Table S2 in Multimedia Appendix 1). Moreover, the average DSCs for the abdominal aorta and bowel segmentation models are 0.906 (95% CI 0.901-0.910) and 0.924 (95% CI 0.913-0.946), respectively, as shown in Table S2 in Multimedia Appendix 1. Furthermore, a comparison of the segmentation masks across the 3 results for 5 patients (Figure 2A-E) indicates that the segmentation model’s outcomes accurately delineate the anatomy of the regions of interest. Additionally, we have made the trained segmentation models available as open source for use in inference and transfer learning [31]. These models were subsequently combined with the MMP risk identification models in the external validation cohort for enhanced risk assessment.

### Performance of the MAM Model

The performance of the MAM model was evaluated via 5-fold cross-validation on the Anzhen cohort and independent external validation on the Gulou cohort, with training strategies and ablation analyses detailed in the Supplemental Methods and Table S3 in Multimedia Appendix 1.

**Figure 3:**
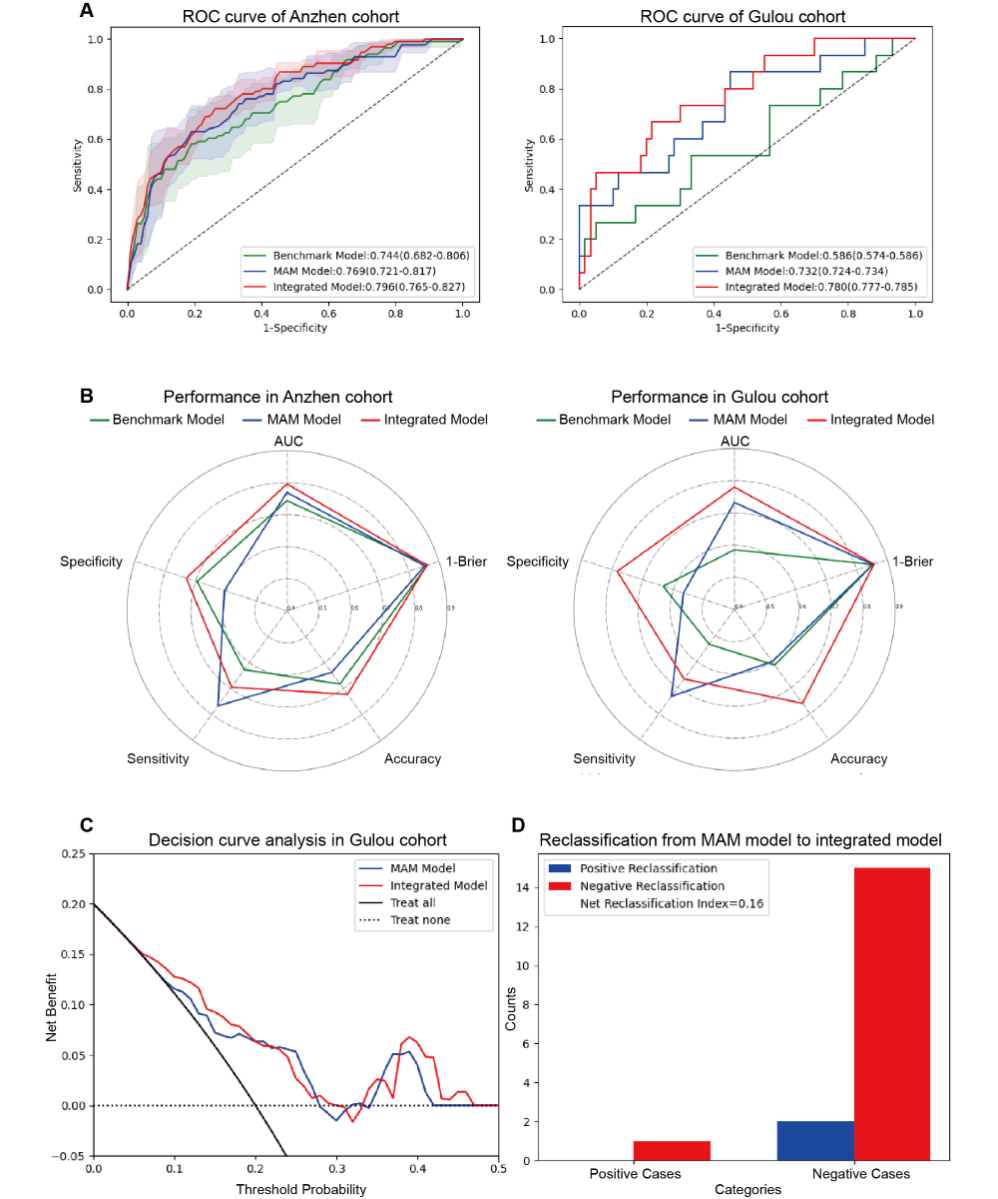
Performance analysis of the models. (A) ROC curves displaying the AUC and 95% CIs for the Anzhen cohort and the Gulou cohort, comparing the multiorgan feature-based acute aortic dissection complicating mesenteric malperfusion (MAM) model, benchmark clinical model, and integrated model. (B) Radar chart visualization depicting a comprehensive performance analysis of the 3 models. (C) Decision curve analysis of the MAM model and integrated model. (D) Net reclassification index comparisons between the MAM model and the integrated model. AUC: area under the curve; ROC: receiver operating characteristic.

According to the ROC analysis (Figure 3A), the MAM model achieved an average AUC of 0.769 (95% CI 0.721-0.817) in the Anzhen cohort and a similar AUC of 0.732 (95% CI 0.724-0.734) in the Gulou cohort. Additionally, using a cutoff determined by the maximum Youden index in the Anzhen cohort, the MAM model exhibited comparable accuracy between the Anzhen cohort (0.638, 95% CI 0.585-0.691) and the Gulou cohort (0.600, 95% CI 0.592-0.600); the sensitivities were 0.767 (95% CI 0.674-0.860) and 0.733 (95% CI 0.721-0.735), with specificities of 0.605 (95% CI 0.543-0.668) and 0.567 (95% CI 0.559-0.567) in the Anzhen and Gulou cohorts, respectively (Table S4 in Multimedia Appendix 1). With respect to model calibration, the model yielded a Brier score of 0.147 (95% CI 0.130-0.164) in the Anzhen cohort and a Brier score of 0.147 (95% CI 0.147-0.149) in the Gulou cohort (Table S4 in Multimedia Appendix 1). Across cohorts, the Gulou cohort showed closely aligned performance, with an AUC of 0.732 versus 0.769, accuracy of 0.600 versus 0.638, sensitivity of 0.733 versus 0.767, and specificity of 0.567 versus 0.605, reinforcing its consistent predictive ability across diverse settings.

### Evaluating and Incorporating Clinicopathological Features

We conducted univariate and multivariate analyses of basic laboratory tests required for diagnosing AAD, and the results revealed that white blood cell (WBC) count (odds ratio [OR] 1.109, 95% CI 1.013-1.214; *P*=.03), neutrophil (NE) count (OR 1.043, 95% CI 1.001-1.088; *P*=.048), D-dimer level (OR 1.012, 95% CI 1.001-1.023; *P*=.049), and lactate level (OR 1.226, 95% CI 1.018-1.478; *P*=.03) were independent predictors of MMP (Table 2). Using these factors, we developed a benchmark clinical model via logistic regression. The model’s AUC values in the Anzhen and Gulou cohorts were 0.744 (95% CI 0.682-0.806) and 0.586 (95% CI 0.574-0.586), respectively (Figure 3A). Additionally, when evaluated at a threshold set by the maximum Youden index from the Anzhen cohort, the model yielded an accuracy of 0.682 (95% CI 0.633-0.731), a sensitivity of 0.627 (95% CI 0.505-0.749), and a specificity of 0.695 (95% CI 0.657-0.734) in the Anzhen cohort, whereas the accuracy was 0.613 (95% CI 0.609-0.616), the sensitivity was 0.533 (95% CI 0.514-0.531), and the specificity was 0.633 (95% CI 0.631-0.638) in the Gulou cohort (Table S4 in Multimedia Appendix 1). For calibration performance, the Brier score was 0.138 (95% CI 0.132-0.143) in the Anzhen cohort and 0.148 (95% CI 0.145-0.149) in the Gulou cohort (Table S4 in Multimedia Appendix 1). Among both cohorts, the benchmark clinical model’s performance declined substantially across key metrics, reflecting a notable reduction in predictive capacity during external validation.

**Table 2:** Univariate and multivariate analyses of risk factors for mesenteric malperfusion.

Characteristics	Univariate analysis			Multivariate analysis	
	OR^a^ (95% CI)	*P* value	OR (95% CI)		*P* value
Age (years)	0.974 (0.952-0.996)	.02	0.982 (0.956-1.009)		.19
White blood cell count, (×109)	1.235 (1.166-1.309)	<.001	1.109 (1.013-1.214)		.03
Neutrophil count, (×109)	1.128 (1.071-1.187)	<.001	1.043 (1.001-1.088)		.048
ALT^b^ (µkat/L)	1.091 (0.936-1.273)	.26	N/A^c^		N/A
AST^d^ (µkat/L)	1.163 (0.999-1.352)	.05	N/A		N/A
Urea (mmol/L)	1.007 (0.998-1.015)	.11	N/A		N/A
Creatine (μmol/L)	1.002 (1.001-1.004)	.01	1.002 (0.999-1.004)		.06
CK-MB^e^ (µg/L)	1.004 (0.999-1.010)	.14	N/A		N/A
Sodium (mmol/L)	1.000 (0.973-1.029)	.98	N/A		N/A
Potassium (mmol/L)	0.769 (0.481-1.229)	.27	N/A		N/A
Protein (g/L)	0.984 (0.952-1.018)	.35	N/A		N/A
Albumin (g/L)	1.008 (0.970-1.047)	.69	N/A		N/A
FDP^f^ (mg/L)	1.003 (1.001-1.006)	.01	0.990 (0.980-1.001)		.07
D-dimer (nmol/L)	1.005 (1.001-1.008)	.006	1.012 (1.001-1.023)		.049
Lactate (mmol/L)	1.525 (1.297-1.792)	<.001	1.226 (1.018-1.478)		.03

^a^OR: odds ratio.

^b^ALT: alanine aminotransferase.

^c^N/A: not applicable.

^d^AST: aspartate aminotransferase.

^e^CK-MB: creatine kinase–MB fraction.

^f^FDP: fibrin degradation products.

To further explore the complementary capabilities of CTA image features and clinicopathological features in terms of predictive performance, we used an attention mechanism for multimodal fusion and retrained an integrated model. Compared with the MAM model, this model showed improved discriminative performance, with AUCs of 0.796 (95% CI 0.765-0.827) in the Anzhen cohort and 0.780 (95% CI 0.777-0.785) in the Gulou cohort (Figure 3B), significantly outperforming the MAM in the external validation cohort (AUC 0.780 vs 0.732, *P*=.02; Figure 3B, Figure S4 in Multimedia Appendix 1). Further evaluation revealed accuracies of 0.722 (95% CI 0.682-0.762) and 0.760 (95% CI 0.758-0.764), and specificities of 0.731 (95% CI 0.676-0.785) and 0.783 (95% CI 0.781-0.788) in the Anzhen and Gulou cohorts, respectively (Table S4 in Multimedia Appendix 1). However, after incorporating clinicopathological features, the sensitivity decreased to 0.695 (95% CI 0.634-0.755) in the Anzhen cohort and 0.667 (95% CI 0.659-0.675) in the Gulou cohort (Table S4 in Multimedia Appendix 1). Furthermore, the integrated model showed generalization comparable with that of the MAM model, with the AUC (0.796-0.780) and specificity (0.731-0.783) shifting modestly from the Anzhen cohort to the Gulou cohort.

Owing to the different time ranges of the Anzhen (2015-2022) and Gulou (2019-2022) cohorts, we evaluated the performance of the integrated model across periods within the Anzhen cohort. The AUCs for early cases (2015-2019, n=248) and recent cases (2020-2022, n=202) were 0.79 and 0.78, respectively, which are consistent with the external validation results (AUC=0.78) (Figure S5 in Multimedia Appendix 1).

Calibration assessment revealed Brier scores of 0.140 (95% CI 0.131-0.149) in the Anzhen cohort and 0.143 (95% CI 0.143-0.145) in the Gulou cohort, outperforming the other models (Table S4 in Multimedia Appendix 1). Additionally, in the Gulou cohort, decision curve analysis indicated that the integrated model provided additional clinical benefits over the MAM model within a probability threshold range of 0.05-0.47 (Figure 3C). Furthermore, the net reclassification index of the integrated model exceeded that of the MAM model by 0.16 (Figure 3D). Specifically, it incorrectly reclassified 2 positive samples as negative and correctly reclassified 15 negative samples from positive to negative predictive classification.

### Model Interpretability Analysis

**Figure 4:**
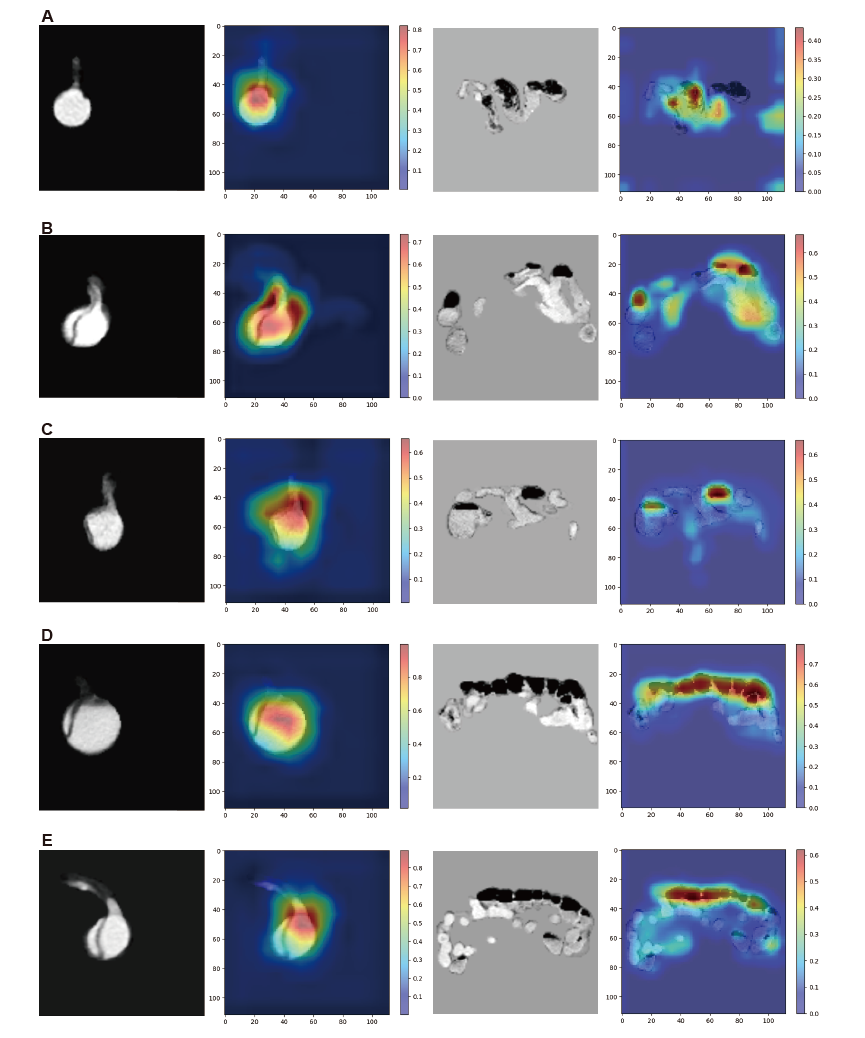
Attention maps of the multiorgan feature-based acute aortic dissection complicating mesenteric malperfusion model using gradient-weighted class activation mapping (Grad-CAM). Panels A-E display computed tomography angiography (CTA) images and attention maps generated by Grad-CAM for 5 randomly selected patients with mesenteric malperfusion. Each panel shows the abdominal aorta CTA cross-section on the left and the bowel CTA cross-section on the right. The red regions indicate high-weight areas critical to model decision-making, whereas the blue regions indicate areas with low weights.

To investigate the regions of interest in deep learning model predictions, we generated attention heatmaps for 5 positive prediction samples, as shown in Figure 4A-E. The results revealed that, in the MAM model, the abdominal aorta encoder and bowel encoder exhibit distinct attention patterns for input CTA images. Specifically, during positive predictions, the encoder processing of abdominal aorta CTA images focuses more on the SMA origin, with heatmaps displaying red regions, indicating that these regions are critical anatomical structures for MMP risk prediction. Moreover, the encoder processing of bowel CTA images concentrates on pathological features such as intestinal dilation, fluid, and air accumulation, resulting in red distributions in heatmaps, indicating high model attention to these regions. The model’s predictive logic aligns with clinical diagnostic thinking for MMP, further confirming its reliability. Additionally, 2 independent risk factors for inhospital mortality were identified among patients undergoing endovascular or surgical treatments, namely, the lactate level (OR 1.255, 95% CI 1.033-1.525; *P*=.02) and the RS (OR 1.030, 95% CI 1.004-1.056; *P*=.02), as shown in Table 3.

**Table 3:** Univariate and multivariate analyses of risk factors for mortality in surgical patients in both cohorts.

Characteristics	Univariate analysis			Multivariate analysis	
	OR^a^ (95% CI)	*P* value	OR (95% CI)		*P* value
Type of AAD^b^ (A vs B)	2.567 (1.201-5.484)	.01	0.490 (0.210-1.144)		.10
Age (years)	0.960 (0.927-0.993)	.02	0.978 (0.940-1.017)		.26
White blood cell count (×10^9^)	1.140 (1.052-1.236)	.001	1.039 (0.942-1.146)		.45
Neutrophil count (×10^9^)	1.038 (1.005-1.071)	.02	1.024 (0.986-1.062)		.21
ALT^c^ (µkat/L)	1.388 (1.124-1.714)	.002	1.277 (0.764-2.134)		.35
AST^d^ (µkat/L)	1.413 (1.130-1.765)	.002	0.899 (0.474-1.704)		.74
Urea (mmol/L)	1.015 (0.998-1.033)	.09	N/A^e^		N/A
Creatine (μmol/L)	1.001 (0.999-1.003)	.45	N/A		N/A
CK-MB^f^ (µg/L)	1.013 (0.993-1.033)	.20	N/A		N/A
Sodium (mmol/L)	1.009 (0.943-1.080)	.80	N/A		N/A
Potassium (mmol/L)	1.122 (0.910-1.385)	.28	N/A		N/A
Protein (g/L)	0.974 (0.920-1.031)	.36	N/A		N/A
Albumin (g/L)	0.979 (0.919-1.043)	.51	N/A		N/A
LDH^g^ (µkat/L)	1.071 (1.011-1.136)	.02	1.040 (0.972-1.112)		.26
D-Dimer (nmol/L)	1.005 (1.000-1.009)	.03	1.004 (0.999-1.009)		.09
Lactate (mmol/L)	1.381 (1.147-1.663)	<.001	1.255 (1.033-1.525)		.02
Risk score (%)	1.028 (1.005-1.051)	.02	1.030 (1.004-1.056)		.02

^a^OR: odds ratio.

^b^AAD: acute aortic dissection.

^c^ALT: alanine aminotransferase.

^d^AST: aspartate aminotransferase.

^e^N/A: not applicable.

^f^CK-MB: creatine kinase–MB fraction.

^g^LDH: lactate dehydrogenase.

## Discussion

### Principal Findings

In this study, we used CTA imaging and clinicopathological data from 525 patients with AAD at 2 centers to develop an integrated model with AUCs of 0.796 and 0.780 in the Anzhen and Gulou sets, respectively. MMP is a severe complication of AAD. This study revealed that the inhospital mortality rate for patients with MMP was approximately 16.67% (15/90), roughly 2 times higher than that for patients with no MMP. The RS (OR 1.030, 95% CI 1.004-1.056; *P*=.02), which was calculated on the basis of the integrated model, was an independent risk factor for mortality in patients with AAD undergoing endovascular or surgical treatment. The preoperative lactate level (OR 1.255, 95% CI 1.033-1.525; *P*=.02) was identified as another independent risk factor affecting the prognosis of MMP, which is consistent with previous reports [7].

The high mortality risk associated with MMP highlights the urgency of early clinical detection. Diagnosis during initial assessment remains notoriously difficult because of insufficient diagnostic evidence and overlapping nonspecific symptoms, such as abdominal pain. In our cohort, 67.78% (61/90) of patients with MMP presented with abdominal pain, closely mirroring the 60% prevalence reported in prior studies [6], thereby reinforcing its limited diagnostic specificity. Furthermore, our data emphasize the high frequency of concurrent malperfusion in other organs among patients with MMP (53/90, 58.89%), which is consistent with existing reports. For example, a systematic review reported that 25% of patients with lower limb ischemia also experience visceral ischemia, 31% of whom have involvement of the SMA [38], whereas Yang et al [7] reported that renal malperfusion was more common in patients with MMP. Similarly, patients with MMP in our cohort presented significantly higher rates of concurrent ischemia in other organs than patients with no MMP did (58.89% vs 22.22%; *P*<.001). Despite the clinical importance of early diagnostic indicators, these parameters rely heavily on subjective symptom interpretation and physical examination, complicating objective quantification. Moreover, quantitative evaluation becomes particularly challenging in unconscious patients, who cannot provide reliable symptom descriptions.

To address the subjectivity of symptoms, laboratory biomarkers offer objective measures for AMI progression. Our analysis confirmed that the WBC count, NE count, lactate level, and D-dimer level are independent predictors of MMP, which is consistent with clinical guidelines [16]. Pathophysiologically, intestinal ischemia impairs mucosal barrier function, promoting bacterial translocation and initiating a systemic inflammatory cascade [39]. This mechanism explains the characteristic elevation of inflammatory markers in intestinal ischemia [40]. Under tissue hypoxia, anaerobic glycolysis predominates, resulting in lactate accumulation and subsequent hyperlactatemia [41]. Lactate is a critical biomarker for the diagnosis of intestinal ischemia and is significantly associated with the risk of intestinal necrosis [10,42]. D-dimer, as a product of fibrin degradation, significantly increases during thrombosis and fibrinolysis [43], demonstrating its diagnostic sensitivity for AMI reaching 82%-96% [44]. However, these markers may also significantly increase in patients with AAD [45-47], substantially compromising their specificity for MMP. Critically, no currently available biomarker achieves sufficient specificity for confirming intestinal ischemia in clinical practice [48]. While emerging biomarkers such as intestinal fatty acid–binding protein, SM22, and exhaled volatile organic compounds await validation [49-51], the integration of multimodal data may represent the most viable strategy to improve diagnostic precision.

Given the limitations of biomarkers, CTA remains a pivotal imaging modality for evaluating AAD with MMP. Prior studies on vascular morphology have indicated that a low true-to-false lumen diameter ratio may predict the risk of developing MMP in patients with AAD with SMA involvement [12]. However, this marker has limited specificity. For example, Zhang et al [52] reported that 19% of patients with isolated SMA dissections were asymptomatic, and 43% demonstrated arterial remodeling following conservative management. These findings suggest that relying exclusively on vascular morphological assessments may not reliably identify MMP. Furthermore, while CTA is effective in detecting advanced signs of bowel ischemia and necrosis, its sensitivity for early ischemic signs such as intestinal dilation, thinning or thickening of the bowel wall, and reduced bowel wall enhancement is limited. The reported sensitivity is approximately 40%, and interobserver agreement is suboptimal [18,53]. Consequently, despite being the preferred imaging method for diagnosing MMP, the use of CTA for detecting early-stage disease remains limited. To increase diagnostic accuracy, integrating CTA findings with clinical indicators is essential.

No specialized tools currently exist for assessing the risk of MMP in patients with AAD. To address this gap, we developed 3 machine learning models: the benchmark clinical model, the MAM model, and the integrated model. In contrast to prior studies that were predominantly concerned with aortic morphology [12], we integrated comprehensive multimodal data to enhance diagnostic performance. Across all cohorts, the integrated model demonstrated the best generalization, achieving AUCs of 0.796 in the Anzhen cohort and 0.780 in the Gulou cohort. In contrast, the AUC of the benchmark clinical model decreased markedly from 0.744 in the Anzhen cohort to 0.586 in the Gulou cohort, highlighting the limitations of laboratory biomarkers in assessing mesenteric ischemia. Furthermore, this substantial decline indicates that models relying solely on simple clinical parameters are limited in addressing overfitting risks stemming from limited sample sizes and imbalanced label distributions. These findings align with those of a prior study on multimodal diagnostic models for AMI, which reported AUCs of 0.72 and 0.60 in the training and testing cohorts, respectively, failing to achieve satisfactory diagnostic performance for mesenteric ischemia [54]. Collectively, these results highlight the superiority of multimodal fusion in reducing cohort variability and affirm the integrated model’s robust generalization capabilities.

The integrated model demonstrated superior diagnostic performance to both the MAM and clinical benchmark models, confirming the efficacy of clinicopathological features in enhancing risk stratification. Notably, although the MAM model maintained high sensitivity, integrating clinicopathological parameters improved specificity at the expense of sensitivity. These changes are likely attributed to the limitations of conventional biomarkers. Specifically, elevated WBC counts, NE counts, and lactate levels often signal progression to conditions such as sepsis and lactic acidosis, reflecting systemic consequences due to advanced ischemia rather than early-stage MMP pathology [55].

While deep learning models highlight their clinical use through enhanced diagnostic accuracy, their black box nature in decision-making processes poses challenges for clinical trust [56]. To address the interpretability limitation, we implemented gradient-weighted class activation mapping to decode critical regions driving model predictions (Figure 4). Visual analysis revealed that the model’s weights were highly concentrated on specific imaging features, which varied depending on the type of input image. Notably, in the abdominal aorta analyses, attention was focused on the abdominal aorta and the SMA origin. Conversely, in bowel image processing, the model highlighted regions suggesting pathological alterations, such as intestinal dilation, air accumulation, and fluid accumulation. This behavioral pattern aligns with the diagnostic logic that clinicians follow when diagnosing MMP [57].

Beyond interpretability, the clinical translation of deep learning models demands efficient standardization of image preprocessing, among which manual VOI annotation emerges as a pivotal challenge because of its labor-intensive nature and interobserver variability [58]. To bridge this critical gap, we developed semantic segmentation models for the abdominal aorta and bowel in CTA images of patients with AAD, creating a fully automated predictive pipeline. The DSC for the abdominal aorta was 0.906, similar to results from previous studies on aortic semantic segmentation models [59]. The DSC for the bowel was 0.924, closely matching the performance of a prior 2-stage framework known as BowelNet [60].

By integrating multiorgan segmentation and risk prediction, our cascaded model enables a fully automated, rapid MMP risk identification workflow, achieving an AUC of 0.780 in external validation, streamlining high-risk patient identification in emergencies. Patients with type A AAD with MMP often face a treatment prioritization dilemma between aortic repair and mesenteric reperfusion [61,62], and early MMP detection facilitates decision-making by guiding preoperative planning. Moreover, the intestine is at risk of irreversible ischemic damage within 6 hours [63], leading to necrosis and mortality. To address this time-critical need, we incorporated essential examinations and laboratory data closely associated with the diagnosis and workflow of AAD, including CTA, WBC, NC, D-dimer, and lactate levels, into the model, optimizing emergency screening and early detection. These improvements potentially transform clinical practices by accelerating triage, refining presurgical planning, and optimizing resource allocation. Furthermore, this automated workflow integrates artificial intelligence with clinical needs, providing radiologists with precise automated segmentation and risk identification to streamline CTA analysis and reduce diagnostic variability [64], assisting surgeons in making treatment decisions on the basis of MMP RSs, and enabling machine learning practitioners to improve models through clinical feedback.

In this study, we aimed to identify patients with AAD who were at high risk of MMP. To enhance clinical use, researchers should validate the model in multicenter prospective cohorts, particularly in Western populations, which would account for demographic diversity, disease heterogeneity, and health care system disparities through international collaboration. Furthermore, recent advances in AAD research have yielded several machine learning models for predicting complications such as aortic rupture [65], acute kidney injury [66], acute ischemic stroke [67], and inhospital mortality [68]. By integrating these models with our MMP risk assessment tool into a unified clinical decision support system, clinicians could achieve preoperative risk stratification in emergency settings. Such integration could enhance patient triage and treatment prioritization and simultaneously streamline resource allocation through data-driven clinical pathways.

### Limitations

This study has several limitations. First, the retrospective design introduces selection biases, for which we implemented strict inclusion and exclusion criteria to mitigate their impact. Second, the multicenter study design may introduce temporal biases. Different data collection periods between the training (2015-2022) and external validation (2019-2022) cohorts potentially reflect evolving clinical practices. While the absence of detailed timing data within the 12-hour window for laboratory tests and CTA imaging collection may introduce variability affecting cohort comparability, given the acute nature of AAD and MMP and the robust model validation, this is unlikely to substantially affect the results. Future prospective validation is needed to eliminate potential temporal biases. Third, since both cohorts are from Chinese urban hospitals, the generalizability of the model to different health care systems, patient demographics, and clinical practices is limited. Further multicenter studies targeting diverse populations are needed to address this limitation. Fourth, the study’s reliance on CTA as the primary imaging modality may introduce variability due to differences in imaging protocols across centers, and the prior imaging of some patients at referring hospitals restricted the availability of digital CTA images. Finally, the extreme caution exercised in clinical practice regarding abdominal exploration and bowel resection surgeries for AAD has led to a paucity of pathological evidence for MMP in most samples. However, we relied on established standards from prior studies and comprehensively evaluated MMP diagnostic criteria from multiple perspectives to minimize this impact.

### Conclusions

This study developed a deep learning model based on multiorgan and multimodal features to identify patients at high risk of developing MMP. If validated in large-scale prospective cohorts, this model could become a rapid diagnostic tool for MMP at the early stages of clinical presentation, thereby reducing mortality risks and informing individualized treatment strategies.

## Data Availability

To request access to the computed tomography angiography images and clinical data reported in this study, contact the lead author. All the original code has been deposited on the GitHub website [31] and is publicly available as of the date of publication. Any additional information required to reanalyze the data reported in this paper is available from the lead author upon request.

## Appendix

Multimedia Appendix 1 Additional methods and results.

## Abbreviations

AAD: acute aortic dissection
AMI: acute mesenteric ischemia
AUC: area under the curve
CTA: computed tomography angiography
DSC: Dice similarity coefficients
IMA: inferior mesenteric artery
MAM: multiorgan feature-based acute aortic dissection complicating mesenteric malperfusion
MMP: mesenteric malperfusion
NE: neutrophil
OR: odds ratio
RS: risk score
SMA: superior mesenteric artery
VOI: volume of interest
WBC: white blood cell
