# Supplementary material for: A Deep Learning Model for Identifying the Risk of Mesenteric Malperfusion in Acute Aortic Dissection Using Initial Diagnostic Data: Algorithm Development and Validation
Source: J Med Internet Res. 2025 Jun 10;27:e72649. doi: 10.2196/72649 (PMC12188138; doi:10.2196/72649)
Supplement: Multimedia Appendix 1 [file jmir_v27i1e72649_app1.docx]

# Supplemental Methods

## Patients

The Anzhen hospital records of 6,032 patients who underwent aortic dissection between January 2015 and June 2022 were retrieved. The inclusion criteria for the MMP group were as follows: (a) availability of clinical pathology data and CTA images in electronic medical records and the picture archiving and communication system (PACS); (b) acute aortic dissection occurring within 14 days of onset; and (c) AD complicated by MMP. The exclusion criteria were as follows: (a) absence of preoperative CTA data (n=33); (b) history of gastrointestinal surgery (n=1); and (c) low-quality images (n=1). Among the remaining 1,532 patients, propensity score matching on the basis of the year of consultation and sex at a ratio of 1:4 yielded 360 negative samples. Ultimately, 450 patients (90 with MMP and 360 without MMP) were included in the Anzhen cohort.

For the Drum Tower Hospital dataset, we retrieved data for 1,582 patients who underwent endovascular or surgical treatment for AAD from January 2019 to December 2022. The same inclusion criteria used for the Anzhen cohort were applied. The exclusion criteria were as follows: (a) absence of preoperative CTA (n=29) and (b) low-quality images (n=2). Ultimately, 75 patients (15 with MMP, 60 with non-MMP) were included in the Gulou cohort. The complete patient inclusion flowchart is available in Supplement Figure 1.

## Development of the Deep Learning Model

The MAM model is a deep convolutional neural network with a dual-tower architecture designed to integrate anatomical information from the abdominal aorta and bowel to predict and diagnose AAD complicated with MMP. Initially, it employs two parallel ResNet10 architectures as encoders (Supplement Figure 3A),^1^ each consisting of a 3D convolution layer, a max pooling layer, four ResNet blocks, and an average pooling layer. Each encoder processes image inputs from a different organ, concatenates the features from these organs, and forwards them to a multilayer perceptron network. To integrate clinicopathological and radiological features, we incorporated an attention mechanism into the MAM model to develop the integrated model.

Owing to the low incidence of MMP, deep learning model training was limited by an imbalanced data distribution and limited training data size. Therefore, we employed image data augmentation techniques and weighted random sampling.^2,3^ Image data augmentation generates new images by applying geometric transformations and introducing noise, thereby increasing the diversity and size of the dataset. Weighted random sampling assigns different class weights to each sample to control sampling probabilities, ensuring a more balanced use of data categories during training and improving model performance. Combining these techniques allows the model to be trained on slightly different datasets each epoch, effectively meeting the needs of deep learning models for rare diseases. In this study, we enhanced 30% of the training data in each epoch via four augmentation strategies: the addition of Gaussian noise, contrast adjustment, rotation, and 3D elastic deformation. Additionally, during the training and validation phases of the cross-validation step, we repeatedly sampled 360 training data points until the sample size was expanded to 500. The sampling weights for positive and negative samples were set at a ratio of 4:1.

We also utilized Med3D's pretrained weights for transfer learning.^4^ Compared with other pretrained models, Med3D use of 3D convolutions enables better capture of spatial information in three-dimensional medical images. These weights are derived from training on large-scale medical imaging datasets, ensuring robust analytical performance in large deep learning models despite limited data availability. Additionally, we implemented L2 regularization, batch normalization, dropout, and early stopping techniques to mitigate the risk of overfitting in our model. The results of the deep learning model ablation studies are shown in Supplement Table 3.

During the model training phase, we employed the binary cross-entropy function to compute training losses, using the AdamW algorithm as the optimizer to minimize these losses. The initial learning rate was set to 10--5, which was dynamically adjusted on the basis of the reduction in loss. All the deep learning models were trained over 150 epochs with a batch size of 16 and deployed on two NVIDIA RTX 4090 GPUs, one Intel(R) Xeon(R) Gold 6326 CPU @ 2.90 GHz, and 256 GB of internal memory.

Using a five-fold cross-validation strategy, the deep learning models were trained five times using data from the Anzhen cohort via the same training approach. An ensemble of predictions from these five models was created through voting. Specifically, to account for outliers and ensure model sensitivity, the second highest predicted probability was selected as the final outcome of the model.^5,6^ The effectiveness of the entire pipeline was subsequently evaluated in the data from the Gulou cohort.

# Supplemental Table

## Table S1. Characteristics of patients in the non- mesenteric malperfusion and mesenteric malperfusion group in the Anzhen cohort.

|  | **Anzhen Cohort(n=450)** | | |  |  | |  |
| --- | --- | --- | --- | --- | --- | --- | --- |
| **Characteristic** | **Non-MMP(n=360)** | **MMP(n=90)** | **P value** | | | **Missing rate** | |
| Age, (year) | 52 (43-57.75) | 48 (41-56) | .04 | | | 0(0.00%) | |
| Sex, (Female) | 32(8.89%) | 8(8.89%) | 1.00 | | | 0(0.00%) | |
| Types of AD |  |  | .13 | | | 0(0.00%) | |
| A | 176(48.89%) | 36(40%) |  | | |  | |
| B | 184(51.11%) | 54(60%) |  | | |  | |
| Mortality | 32(8.89%) | 15(16.67%) | .03 | | | 0(0.00%) | |
| Abdominal symptoms | 81(22.5%) | 61(67.78%) | <.001 | | | 0(0.00%) | |
| Other organs ischemia | 80(22.22%) | 53(58.89%) | <.001 | | | 0(0.00%) | |
| WBC, (×10^9^) | 10.47 (7.76-13.48) | 15.3 (11.87-18.27) | <.001 | | | 0(0.00%) | |
| NE, (×10^9^) | 8.66 (5.48-11.77) | 13.32 (9.83-16.44) | <.001 | | | 0(0.00%) | |
| ALT, (µkat/L) | 0.37 (0.25-0.55) | 0.43 (0.31-0.89) | .002 | | | 6(1.33%) | |
| AST, (µkat/L) | 0.33 (0.27-0.48) | 0.52 (0.32-0.99) | <.001 | | | 7(1.56%) | |
| Urea, (mmol/L) | 6.06 (4.91-8) | 7.1 (5.55-8.89) | .001 | | | 3(0.67%) | |
| Creatine, (μmol/L) | 79.3 (67.3-97.75) | 91.9 (71.85-133.8) | .001 | | | 3(0.67%) | |
| CK-MB, (µg/L) | 1.6 (0.9-2.9) | 2 (1.3-4.4) | .004 | | | 15(3.33%) | |
| LDH, (µkat/L) | 3.46 (2.89 -4.34) | 4.53 (3.44 -6.74) | <.001 | | | 117(26.00%) | |
| Sodium, (mmol/L) | 138.8 (136.7-140.5) | 138.5 (135.5-141) | .32 | | | 5(1.11%) | |
| Potassium, (mmol/L) | 3.87 (3.54-4.18) | 3.84 (3.48-4.22) | .49 | | | 5(1.11%) | |
| Protein, (g/L) | 66.05 (61.8-70.23) | 64.9 (61-70.15) | .36 | | | 7(1.56%) | |
| Albumin, (g/L) | 39.2 (36.2-42.5) | 39.6 (36.55-43) | .79 | | | 7(1.56%) | |
| FDP, (mg/L) | 10.28 (4.1-31.73) | 20.25 (11.34-49.11) | <.001 | | | 7(1.56%) | |
| D-Dimer, (nmol/L) | 5.86 (2.74-14.68) | 2.02 (11.06-25.63) | <.001 | | | 3(0.67%) | |
| Lactate, (mmol/L) | 1.4 (1-2) | 2.1 (1.1-3.48) | <.001 | | | 23(5.11%) | |

Values are presented as median (Q1-Q3) for continuous variables and number (%) for categorical variables. AD, acute aortic; WBC, white blood cell count; NE, neutrophil count; ALT, alanine aminotransferase; AST, aspartate aminotransferase; CK-MB, creatine kinase–MB fraction; LDH, lactate dehydrogenase; FDP, fibrin degradation products.

## Table S2. Dice similarity coefficient analysis of volume of interests reproducibility and volume of interest automatic segmentation models.

|  | Reader 2 |  | nnU-Net |  |
| --- | --- | --- | --- | --- |
|  | Abdominal aorta (±SD) | Bowel (±SD) | Abdominal aorta (95%CI) | Bowel (95%CI) |
| Reader 1 | 0.896(±0.026) | 0.907(±0.072) | 0.906(0.901,0.910) | 0.924(0.913,0.946) |

SD, Standard deviation; CI, confidence interval.

## Table S3. Ablation analysis for optimizing the MAM model.

| Hyperparameters | Setup 1 | Setup 2 | Setup 3 | Setup 4 | Setup 5 |
| --- | --- | --- | --- | --- | --- |
| Data augmentation | × | √ | √ | √ | √ |
| Weighted random sampler | × | √ | √ | √ | √ |
| Weight decay | × | × | √ | √ | √ |
| Dropout | × | × | √ | √ | √ |
| Batch normalization | × | × | √ | √ | √ |
| Transfer learning | × | × | × | √ | √ |
| Input modality | Image | Image | Image | Image | Image +Text |
| AUC | 0.667 | 0.642 | 0.724 | 0.752 | 0.83 |

AUC, area under the curve

## Table S4. Model performance assessment and comparison.

|  | Benchmark clinical model | MAM model | Integrated model |
| --- | --- | --- | --- |
| Anzhen cohort |  |  |  |
| AUC (95%CI) | 0.744(0.682-0.806) | 0.769(0.721-0.817) | 0.796(0.765-0.827) |
| ACC (95%CI) | 0.682(0.633-0.731) | 0.638(0.585-0.691) | 0.722(0.682-0.762) |
| Sen (95%CI) | 0.627(0.505-0.749) | 0.767(0.674-0.860) | 0.695(0.634-0.755) |
| Spe (95%CI) | 0.695(0.657-0.734) | 0.605(0.543-0.668) | 0.731(0.676-0.785) |
| Brier (95%CI) | 0.138(0.132-0.143) | 0.147(0.130-0.164) | 0.140(0.131-0.149) |
| Gulou cohort |  |  |  |
| AUC (95%CI) | 0.586(0.574-0.586) | 0.732(0.724-0.734) | 0.780(0.777-0.785) |
| ACC (95%CI) | 0.613(0.609-0.616) | 0.600(0.592-0.600) | 0.760(0.758-0.764) |
| Sen (95%CI) | 0.533(0.514-0.531) | 0.733(0.721-0.735) | 0.667(0.659-0.675) |
| Spe (95%CI) | 0.633(0.631-0.638) | 0.567(0.559-0.567) | 0.783(0.781-0.788) |
| Brier (95%CI) | 0.148(0.145-0.149) | 0.147(0.147-0.149) | 0.143(0.143-0.145) |

AUC, area under the curve; ACC, accuracy; Sen, sensitivity; Spe, specificity; Brier, Brier score; CI, confidence interval.

# Supplemental Figures

## Figure S1. Flowchart of The Inclusion and Exclusion criteria.


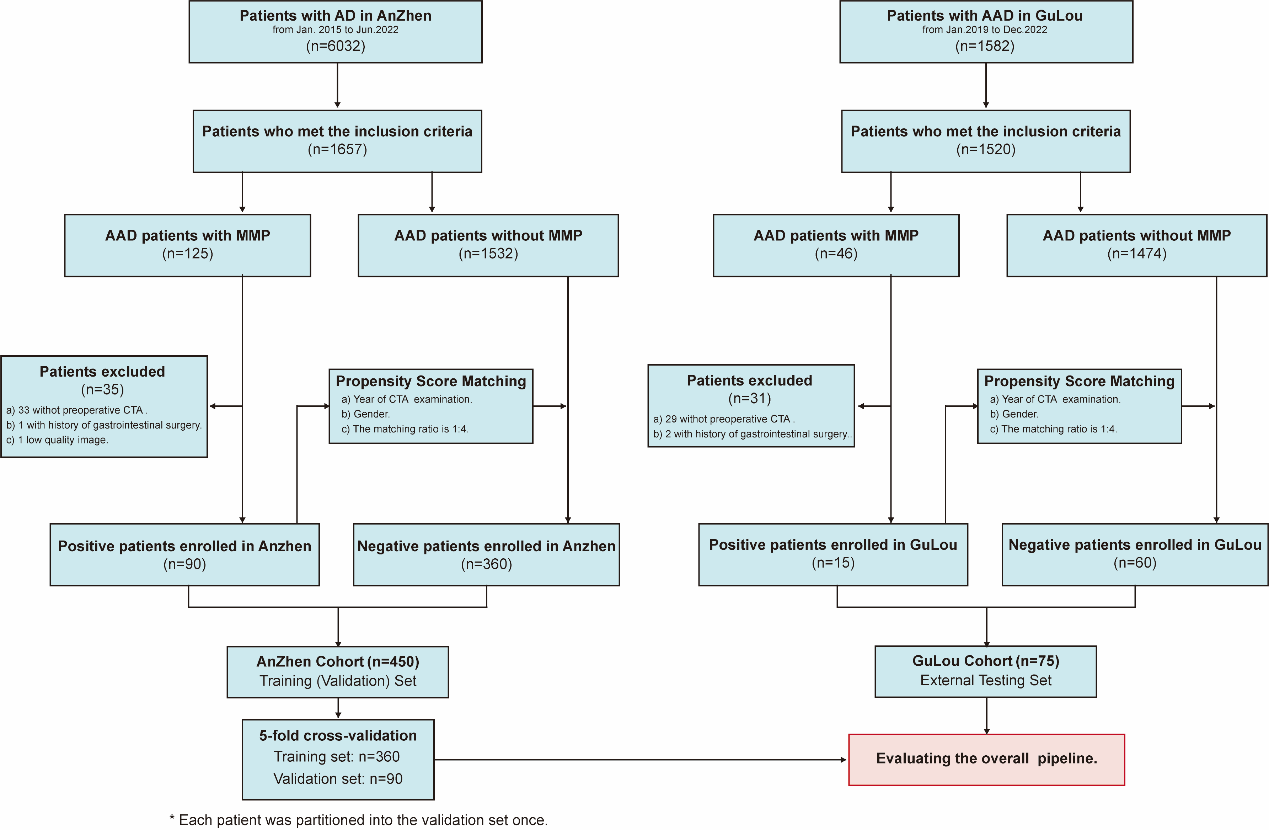


AAD, acute aortic dissection; MMP, mesenteric malperfusion.

## Figure S2. Preprocessing Pipeline for CTA images.


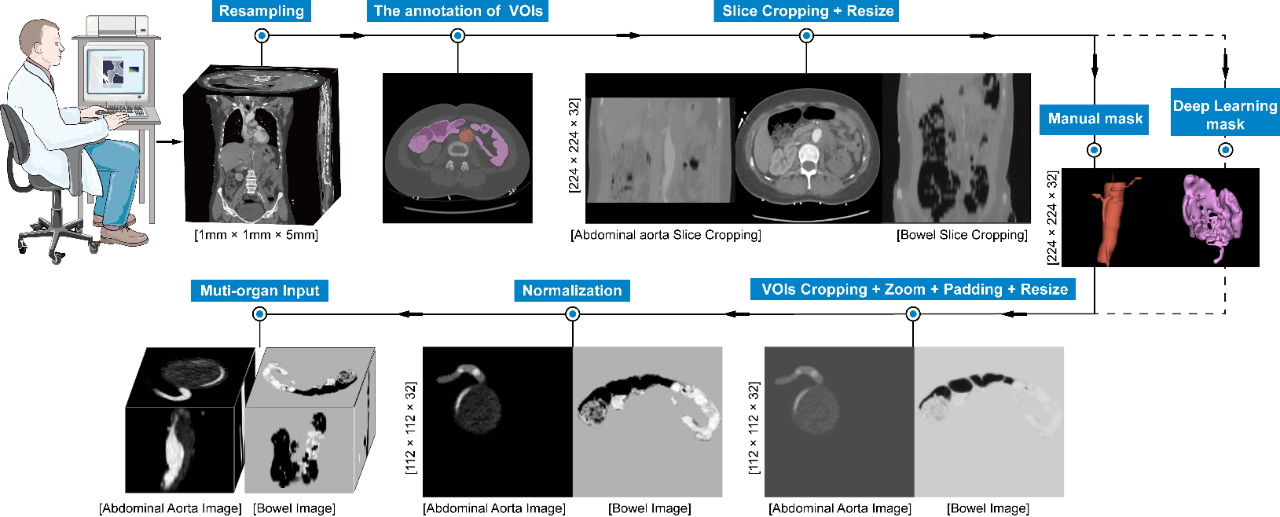


VOI, volumes of interest.

## Figure S3. Deep Learning Model Architecture.


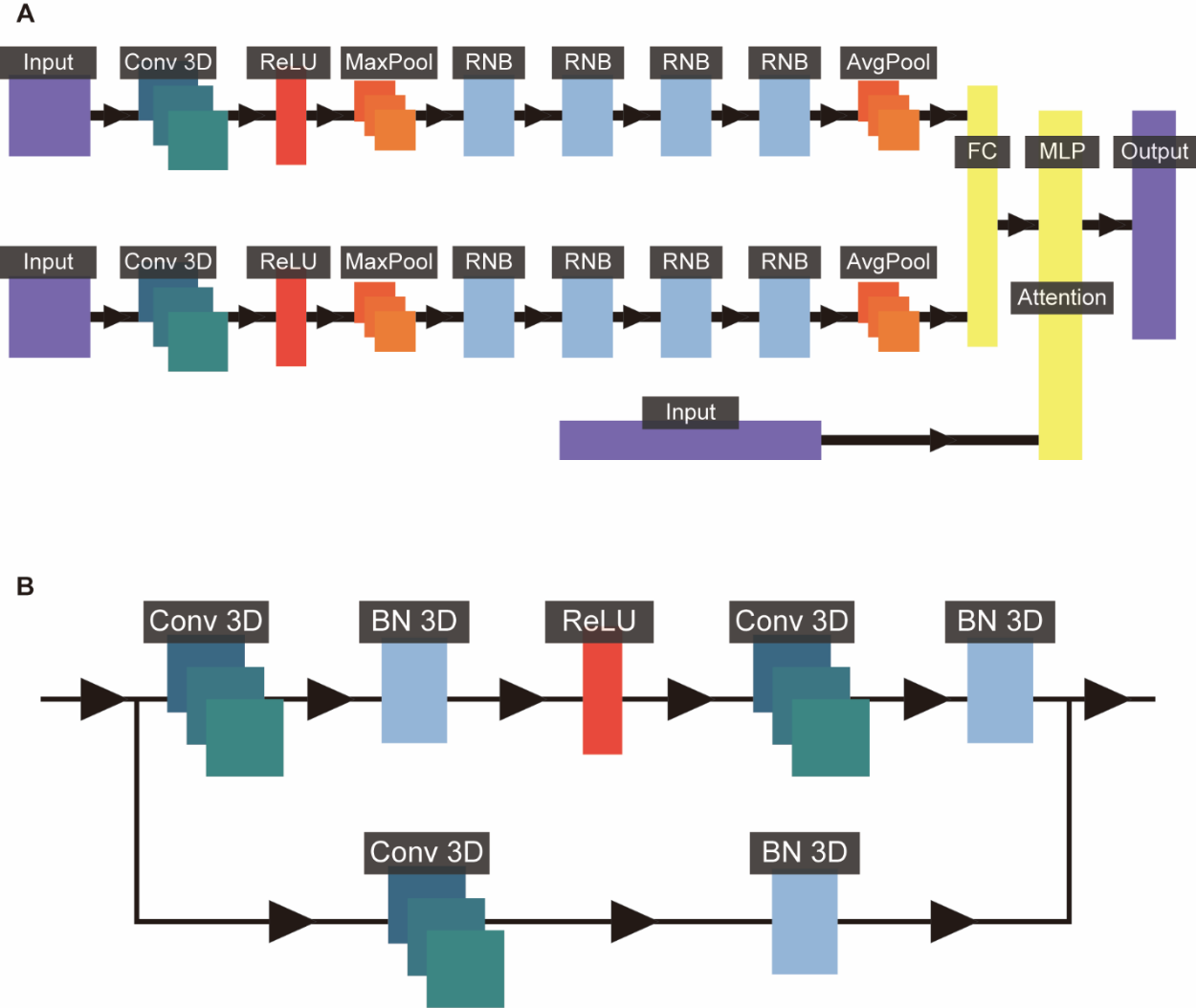


(A) Structure of the integrated model

(B) Structure of the ResNet block (RNB). 3D Convolution (Conv 3D), rectified linear unit (ReLU), maximum pooling (Maxpool), average pooling (Avgpool), fully connected layer (FC), multilayer perceptron (MLP), 3D batch normalization (BN 3D).

## Figure S4. Delong Test Results for MMP risk identification models.


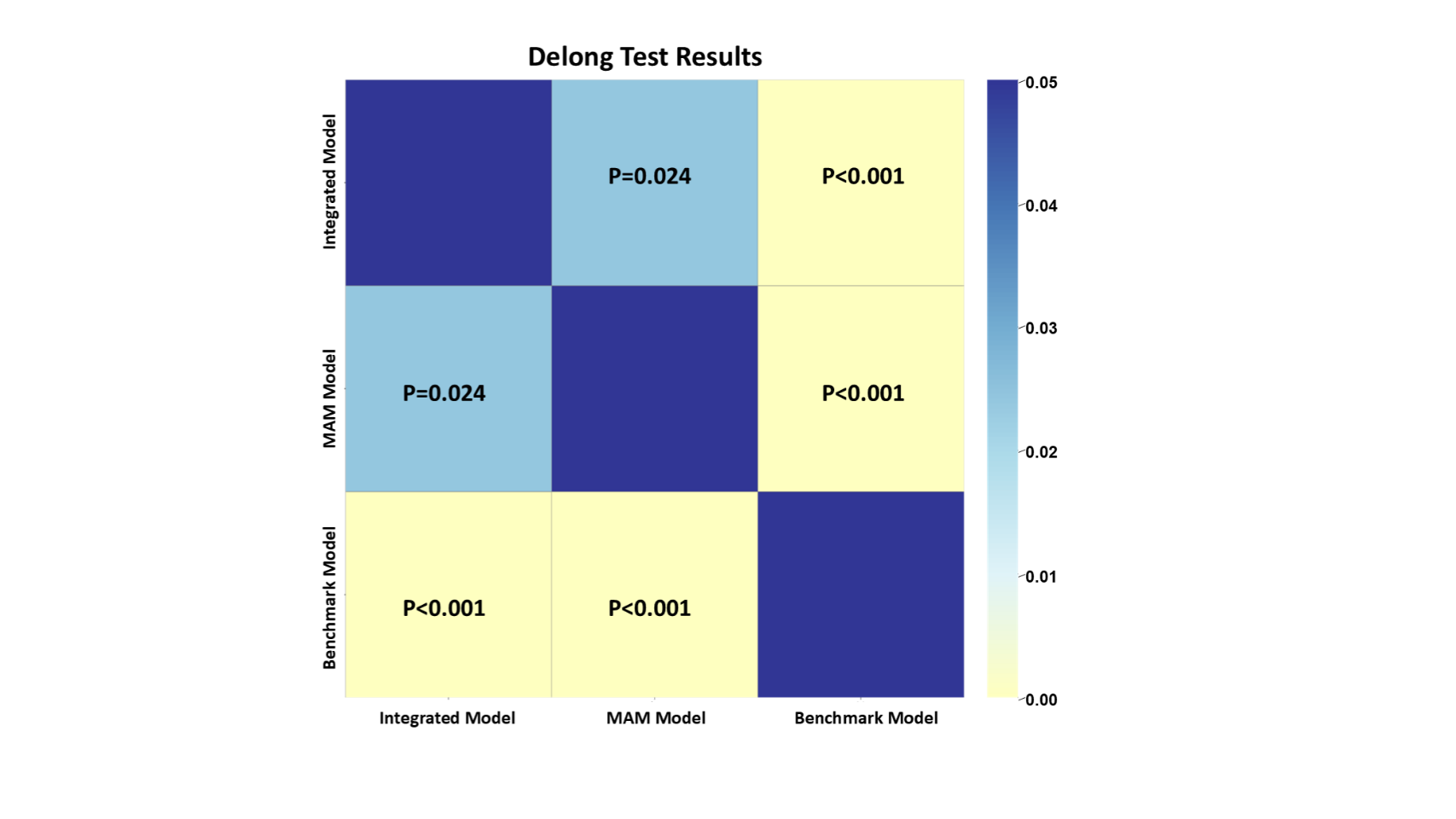


## Figure S5. ROC Curve of the Integrated Model Across Different Enrollment Year Cohorts.


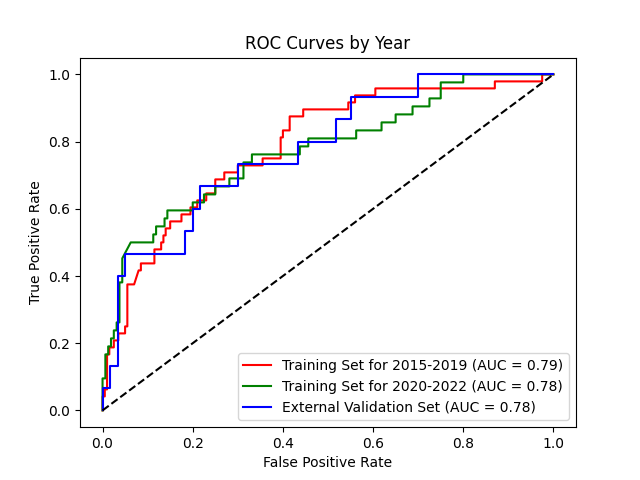


ROC, receiver operating characteristic; AUC, area under the curve.

# Supplemental References

1. K. He, X. Zhang, S. Ren and J. Sun, "Deep Residual Learning for Image Recognition," 2016 IEEE Conference on Computer Vision and Pattern Recognition (CVPR), Las Vegas, NV, USA, 2016, pp. 770-778, doi: 10.1109/CVPR.2016.90.

2. Zhang J, Wu J, Zhou XS, Shi F, Shen D. Recent advancements in artificial intelligence for breast cancer: Image augmentation, segmentation, diagnosis, and prognosis approaches. Semin Cancer Biol. 2023;96:11-25. doi:10.1016/j.semcancer.2023.09.001

3. Efraimidis PS, Spirakis PG. Weighted random sampling with a reservoir. Information Processing Letters. 2006;97(5):181-185. doi:10.1016/j.ipl.2005.11.003

4. Chen S , Ma K , Zheng Y .Med3D: Transfer Learning for 3D Medical Image Analysis. 2019. doi:10.48550/arXiv.1904.00625.

5. Dietterich TG. Ensemble Methods in Machine Learning. In: Multiple Classifier Systems. Vol 1857. Lecture Notes in Computer Science. Springer Berlin Heidelberg; 2000:1-15. doi:10.1007/3-540-45014-9_1

6. Moor M, Bennett N, Plečko D, et al. Predicting sepsis using deep learning across international sites: a retrospective development and validation study. EClinicalMedicine. 2023;62:102124. Published 2023 Aug 11. doi:10.1016/j.eclinm.2023.102124
